# Inhibition of p53 expression modifies the specificity of chromatin binding by the androgen receptor

**DOI:** 10.18632/oncotarget.449

**Published:** 2012-02-29

**Authors:** Natalya V. Guseva, Oskar W. Rokhlin, Thomas B. Bair, Rebecca B. Glover, Michael B. Cohen

**Affiliations:** ^1^ Department of Pathology, University of Iowa Hospitals and Clinics, Iowa City, Iowa, USA; ^2^ DNA Facility, University of Iowa, Iowa City, USA

**Keywords:** Androgen receptor, p53, FoxA1, prostate cancer

## Abstract

The androgen receptor (AR) is known to play a critical role in prostate cancer (PC). p53 likely also plays a role given that p53 mutations are commonly found in advanced PC, and loss of wild-type protein function contributes to the phenotype of castration-resistant prostate cancer (CRPC). Nevertheless, the extent of the contribution of p53 dysfunction to PC remains unclear. Here we analyze the effects of p53 inhibition in PC cells and show that it has significant consequences for both the interaction between AR, and chromatin and the proliferative capacity of these cells. Inhibition of p53 expression enabled LNCaP cells to proliferate independently of androgens. Moreover, it modified the genome-wide binding pattern of AR. ChIP-sequnce analyis (ChIP-seq) revealed that fewer AR-binding sites were present in the context of p53 inhibition, suggesting that wild-type p53 is required for stable binding of AR to certain chromatin regions. Further analysis revealed that a lower AR occupancy was accompanied by a reduction in FoxA1 binding at regulatory regions of AR-dependent genes. Our study also identifies a pool of genes that may be transcriptionally regulated by AR only in the absence of p53, and that may contribute to the CRPC phenotype. Overall, our results point to p53 playing an important role in regulating AR activity across the genome.

## INTRODUCTION

Androgen deprivation therapy is currently the standard treatment for locally advanced, recurrent, or metastatic prostate cancer (PC). However, most patients with metastatic carcinomas progress to a state of disease insensitive to androgen ablation, called castration resistant prostate cancer (CRPC) [1, 2]. The systemic treatment options for CRPC are limited, largely because of our poor understanding of the molecular underpinnings of this disease state. The majority of CRPCs continue to express the androgen receptor (AR) and are dependent on AR signaling for malignant progression [3-5]. p53 is also thought to play a role in PC progression, although the extent of its contribution is poorly understood. Mutations of p53 are uncommon in primary PC, but occur quite frequently in advanced disease [6, 7]. In fact, a recent study has suggested that mutations in p53 lead to CRPC; Burchardt et al., (2001) [8] showed convincingly that a reduction in p53 expression or function in LNCaP confers the ability to form tumors in castrated male nude mice (parental LNCaP cells do not have this ability). In addition, AR and p53 can mutually regulate one another [9, 10]. Both p53 and AR are transcription factors and can affect the expression of a broad array of genes. It is also possible that an alteration in p53 affects transcriptional function of AR, and vice versa.

AR is a ligand-dependent transcription factor. In the absence of androgen binding, it is expressed diffusely throughout the cytoplasm, and held in an inactive state through its association with chaperones such as heat shock proteins (HSPs). Androgen binding releases AR from HSPs, facilitating AR homodimerization and nuclear translocation. Activated AR dimers then bind to DNA at specific sequences termed “androgen-response elements” (AREs) within target-gene regulatory regions. Here they serve as a platform for the recruitment of co-activators and the basal transcriptional machinery, modulating gene expression to promote diverse biological outcomes. Transcription factors also have the ability to regulate gene expression by tethering to other response elements through protein-protein interaction with other DNA-bound factors. Thus, AR might indirectly bind to non-ARE sequences in the genome, through a tethering mechanism that involves other transcription factors such as AP-1 and Sp1 as was shown for estrogen receptor [11, 12]. Thus, the control of AR target genes involves long-range combinatorial regulation of: AR, DNA-binding collaborating transcription factors, and non-DNA binding co-regulatory factors [13-15].

CRPCs were recently reported to feature an altered expression program (with respect to parental PCs), accompanied by a novel AR cistrome [16], which is highly enriched for the FKH motif [14, 17]. FoxA1, also known as hepatocyte nuclear factor HNF-3-alpha, is a key partner for ERα and AR in promoting transcriptional activity in breast and prostate cancer, respectively, and recognizes the FKH motif. FoxA1 interacts with the AR in PC cells, where it influences the regulation of AR target genes [18] by acting as a pioneer factor in binding to nucleosomal DNA. FoxA1 differential binding to chromatin sites is dependent on the distribution of histone H3 lysine 4 (H3K4) dimethylation. Increased H3K4 dimethylation may thus lead to increased recruitment of FoxA1, which in turn would facilitate AR occupancy [16].

To understand the role of p53 in so called “androgen-independent” cell growth of PC, we evaluated the effects of p53 inhibition on the genome-wide chromatin localization of AR in LNCaP. We found that in the absence of p53, the number of AR-binding peaks is significantly reduced, and also that some of those peaks are unique.

## RESULTS

### p53 inhibition interferes with PC cell proliferation and with binding of the AR to DNA

**Figure 1:**
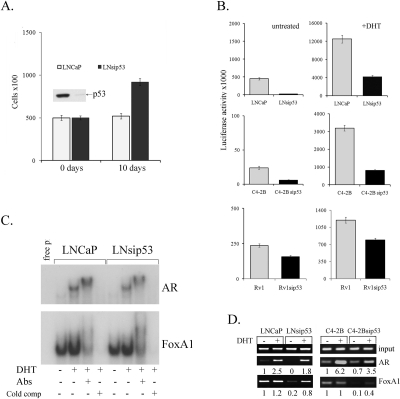
Effects of p53 inhibition on proliferation of LNCaP and on the specificity of chromatin binding by AR A) Bar graph depicting the effects of p53 inhibition on androgen-independent proliferation in LNCaP. Error bars represent mean ±SEM, where n=3. Day 0 expression of p53 in LNCaP and LNsip53 cells was examined by western blot analysis, as shown above the 0-day bars. B) Bar graphs depicting effects of p53 inhibition on AR reporter activity in three distinct prostate cancer lines (LNCaP, C4-2B, and Rv1) stably transfected with an ARE-Luc construct, as assessed by luciferase assay. Error bars represent mean ±SEM, where n=3. C) EMSA assessing the effects of p53 inhibition on binding of AR and FoxA1 to DNA, in lysates of androgen-deprived LNCaP and LNsip53 cells treated with 10 nM of DHT. The ^32^ P-end labeled probe for AR (upper panel) was an ARE, and that for FoxA1 (lower panel) was TTS (FoxA1 responsive element). Image shown is representative of three replicate experiments. D) ChIP-PCR assessing the effects of p53 inhibition on occupancy of the probasin promoter (PB) by AR and FoxA1, in both LNCaP and C4-2B. Anti-AR and anti-FoxA1 antibodies were used for IP, and primers specific for the PB promoter were used for PCR (-242/-100). Images shown are representative of three replicate experiments.

To investigate the role of p53 in the proliferation of PC cells, we used LNCaP that were stably transfected with sip53 (LNsip53) or siE6 (LNsiE6), as previously described [10]. It is well established that LNCaP cells do not proliferate if cultured in charcoal-stripped (CSS) medium, which lacks androgens. We plated the same number of LNCaP control and LNsip53 cells in CSS medium and, after 10 days, detected a 1.5-2.0 fold increase in cell number for LNsip53, but not control cells (Fig. 1A). p53 expression at day 0 was verified by western blot analysis, as shown above the 0-day column in Fig. 1A; p53 expression was undetectable in the LNsip53 cells. To examine the influence of p53 inhibition on AR activity, we used cells permanently transfected with a luciferase reporter under control of the probasin (PB) promoter. In this case, not only LNCaP (LNCaP-ARE-Luc, LNsip53-ARE-Luc) was used, but also C4-2B-ARE-Luc, C4-2Bsip53–ARE-Luc and 22Rv1-ARE-Luc, 22Rv1sip53-ARE-Luc. LNCaP is the most common and well characterized prostate cell line and exhibit many of the properties of androgen dependence seen in clinical disease. In addition to LNCaP only few cell lines expressing AR protein have been described and almost all of them express wild type of p53. In addition to LNCaP we used bone metastatic LNCaP derivative C4-2B, and the CWR22 androgen independent derivative Rv1 cell line that express both full length and truncated isoform of AR. We also considered using MDA-PC-2b derived from a bone metastases and which express both AR and p53. However we could not obtain viable cells after transfection experiments.

Cells from each of these lines were cultured in CSS for three days, and were then either left untreated or treated with 10 nM DHT for 24 hours. As shown in Fig. 1B (top left panel), basal levels of AR activity were significantly lower in LNsip53-ARE-Luc than in LNCaP-ARE-Luc cells. Although DHT treatment induced AR activity in both cell lines, luciferase activity was 5 times lower in the LNsip53-ARE-luc line (Fig. 1B top right panel), which may be related to low background in these cells. The same trend was observed in C4-2B and 22Rv1 cells (Fig. 1B, lower panels). We thus suggest that inhibiting p53 expression minimizes the luciferase response in PC cells by changing AR-mediated transcriptional activation

Given that FoxA1 is a critical facilitator of DNA binding by nuclear receptors, we examined the binding of FoxA1 to DNA in the same cell lines. First, we carried out an electromobility shift assay (EMSA) to compare AR and FoxA1-DNA binding to oligonucleotides containing classical AREs or TTS (FoxA1 responsive element). As shown in Fig. 1C, there were no significant differences in the *in vitro* DNA binding of either AR or FoxA1 between LNCaP and LNsip53, before or after DHT treatment. Likewise, FoxA1 bound to the classical response element in the TTS oligonucleotide probe in both sets of untreated cells. Pretreatment of the samples with anti-AR or anti-FoxA1 antibody, however, affected DNA-protein mobility: the application of anti-AR yielded a supershifted band; the application of anti-FoxA1 resulted in significantly diminished DNA binding. These results confirm that the binding observed was specific.

We next carried out ChIP analysis to investigate the binding of AR and FoxA1 to the PB promoter on our luciferase reporter. To this end, we treated LNCaP-ARE-Luc control, LNsip53-ARE-Luc, C4-2B-ARE-Luc or C4-2Bsip53-ARE-Luc cells with DHT after 3 days of culture in CSS, and performed ChIP using AR or FoxA1 antibodies. We then performed PCR using primers specific to the PB region of this plasmid and quantitated the results by densitometry. As shown in Fig 1D, the levels of PCR products from AR-ChIP were higher in LNCaP than in LNsip53 (left panel); this difference was even greater when the cells were treated with DHT. The results for the FoxA1-ChIP samples were essentially the same. Moreover, the same patterns were found for AR- and FoxA1-ChIP in C4-2B and C4-2Bsip53 (Fig. 1D, right panel). Therefore, the lower occupancy of the PB promoter by FoxA1 and AR in LNsip53-Luc and C4-2Bsip53-Luc cells appears to account for the reduced transcription observed in these contexts (luciferase assay, Fig. 1B), and this phenomenon appears to be common to multiple PC lines. The difference in the binding of both AR and FoxA1 to DNA in the presence (LNCaP) vs. the absence (LNsip53) of p53 suggests that these cells have epigenetic differences.

### Inhibition of p53 expression changes genome-wide patterns of AR binding to DNA

**Figure 2:**
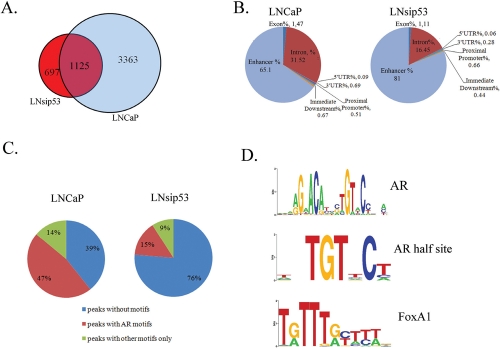
ChIP-Sequence analysis of AR binding sites in LNCaP and LNsip53 (A) Venn diagram showing overlap between the AR binding sites identified in LNCaP and LNsip53 by ChIP-Seq analysis. Peaks meeting the default threshold score of 13 using the Partek GS peakfinding software were aligned based on matches in genomic coordinates. (B) Pie chart indicating relative enrichment of ChIP regions in genomic regions of potential importance, including as promoters, sequences immediately downstreams of genes, and introns. This analysis was carried out for peaks identified in (A), and CEAS was used for annotation. (C) Pie charts showing the percentages of peaks in LNCaP and LNsip53 that contain an AR or other motif (as identified using CEAS). (D) LOGOs (graphical representation of the sequence conservation of nucleotides in a strand of DNA) derived by motif enrichment analysis in AR-bound regions of DNA. The most commonly identified (by CEAS) motifs among the predicted peaks were: the classical ARE binding site, AR-half sites, and FoxA family response elements. All identified LOGOS are listed in Figures S1 and S2.

To determine whether eliminating wild type p53 affects the binding of AR to DNA throughout the genome, we performed ChIP-sequence analysis (ChIP-seq). We obtained 12,331,952 and 11,788,138 DNA sequence reads for LNCaP and LNsip53, respectively. These reads were mapped to the human genome (hg18) and corresponded to 4488 [3363 + (1125)] and 1822 [697 + (1125)] unique peaks, respectively (Fig. 2A). Of these, 1822 represented AR binding sites in LNsip53, 1125 (62%) of which overlapped with those identified in control LNCaP cells (Fig. 2A), demonstrating that some aspects of the AR binding profile are shared in the two cell lines, but that p53 inhibition also leads to AR binding at unique genomic positions.

In both cell lines, the majority of peaks were located in either the intron of a known gene or an intergenic region located more than 10 kb from the nearest gene (Fig. 2B). Notably, fewer intronic peaks were found in LNsip53 than LNCaP. Also, fewer than 1% of the peaks localized to a proximal promoter or 5'-untranslated region of a known gene, consistent with previously published data for AR [19]. Therefore, long-range, AR-mediated regulation of genes from distal sites is most likely a common mechanism in AR-positive PCs.

We next analyzed motif enrichment in AR-bound regions of DNA, using the cis-regulatory element annotation system (CEAS) [20]. Of the 4488 AR-specific binding sites identified in LNCaP cells, 2105 (47%) contained AREs and 618 (14%) contained response elements for other transcription factors but no AREs (Fig. 2C). In LNsip53, only 265 of 1810 (15%) peaks contained AREs (Fig. 2C). All identified peaks are described in Table S1 (LNCaP) and Table S2 (LNsip53). As expected, the most common motifs in both cell lines were: classical ARE binding sites, AR-half sites and FoxA family response elements (Fig. 2D and Table S3). However, additional response elements were also found specifically in the in LNCaP-AR set, for example FoxO1, FoxO3, Freac-2, HFH4, GATA. (Table S3. All motif LOGOS for LNCaP and LNsip53 are listed in Figures S1 and S2, respectively).

### Analysis of AR binding peaks shared by LNCaP and LNsip53 cells

**Figure 3:**
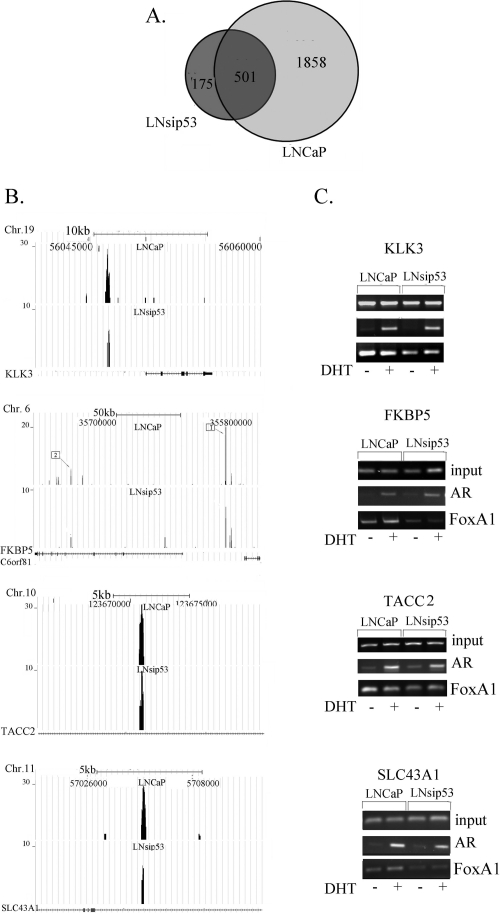
Mapping of ChIP-enriched signals to predicted genes and validation of the interaction (A) Venn diagram showing overlap between the AR-regulated genes in LNCaP and LNsip53 cells. Peaks from Fig 2A were mapped to genes using CEAS. (B) ChIP-seq analysis of the peaks that are shared by the two datasets and that correspond to known target genes. Visualization of AR binding to previously reported target genes KLK3 (PSA), FKBP5, TACC2, and SLC43A1, was performed using UCSC Genome Browser images. (C) ChIP-PCR confirming that AR binds to the identified genes.

ChIP-seq or ChIP-on-ChIP analysis of LNCaP cells was previously reported [16, 21]. We have compared peaks for LNCaP detected in our analysis with those from Yu et al, 2010 and found 75% overlap. Using CAES, we mapped the regions identified by ChIP to the nearest genes; 2359 and 676 genes (listed in Tables S4 and S5) were present near peaks for the LNCaP and LNsip53, respectively (Fig. 3A). The percentage of peaks identified near genes with respect to the number of total peaks was lower in LNsip53 (37, 3%) vs. LNCaP (54, 6%) cells. Comparison of the identified genes revealed that 501 (74%) of those found in LNsip53 were also found in LNCaP (shared group); 175 (26%) genes were unique to LNsip53 cells.

The majority of genes from the shared group had previously been reported as AR regulated. We found AR binding peaks near known AR regulated genes such as KLK3, KLK2, FKBP5, TACC2 and SLC43A1, all of which are reported to be AR-regulated. In both cell lines, AR-specific peaks were found in the enhancer region of KLK3, an upstream enhancer and an intron of FKBP5, and an intron of TACC2 and SLC43A1 (Fig. 3B). We confirmed binding at these sites by ChIP-PCR, using anti-AR and anti-FOXA1 antibody and primers for specific regions (Fig. 3C). The mRNA level of FKBP5 and KLK3 dramatically increased after 8 and 24 hours of DHT treatment for both cell lines (data not shown). Thus, we did not find differences in gene expression in overlapping group.

### Analysis of LNCaP specific AR-binding peaks

**Figure 4:**
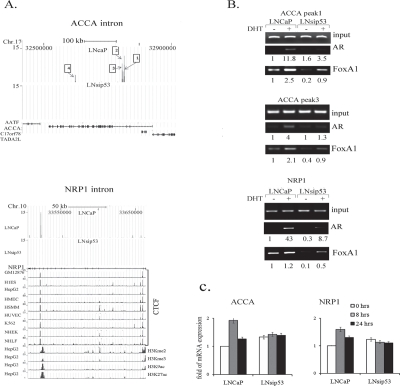
Analysis of LNCaP-specific AR-binding peaks in ACCA and NRP1 genes (A) ChIP-seq defined four peaks of LNCaP-specific binding of AR to introns within the ACCA gene, and to one intron of the NRP1 gene. Comparison to encode histone modes trek in the Genome Browser reveal that the AR binding peak in the NRP1 gene overlaps with the CTCF-binding peaks in a broad spectrum of cell lines (shown on the left). (B) ChIP-PCR confirming binding of AR and FoxA1 to ACCA and NRP1 introns in LNCaP. (C) Semi-quantitative RT-PCR of ACCA and NRP1, revealing mRNA levels after DHT treatment. mRNA levels for ACCA (left panel) and NRP1 (right panel) were calculated with respect to GAPDH; levels in untreated LNCaP cells were arbitrarily defined as equivalent to 1. Error bars represent mean ±SD, where n=3.

Of the list of LNCaP-specific genes, we examined Acetyl-CoA-carboxylase-α (ACCA) and neuropilin-1 (NRP1). In the case of AACA, we found 4 peaks within intronic regions (Fig. 4A, numbered from right to left starting at the beginning of the gene). Analysis of the DNA sequences corresponding to the peaks revealed that each peak contained multiple AR and FoxA1 response elements (Table S6). We verified all 4 peaks in LNCaP using ChIP-PCR (with anti-AR antibody) [22].

The NRP1 gene had one peak specific to LNCaP (Fig. 4B). We compared this peak to enrichment of the H3K4Me2, H3K4Me3, H3K9ac, and H3K27ac histone marks across the genome of HepG2 cells, available on the UCSC browser. We chose to assess the levels since these marks are associated with transcriptional initiation and opening of chromatin structure. We also compared it to the mark of the CTCF zinc finger transcription factor in 8 cell lines featured in the UCSC browser. CTCF is a sequence-specific DNA binding protein that functions as an insulator, blocking enhancer activity. Depending on the cellular context, CTCF can bind a histone acetyltransferase (HAT)-containing complex and function as a transcriptional activator, or bind a histone deacetylase (HDAC)-containing complex and function as a transcriptional repressor. We found that the AR peak in NRP1 in LNCaP overlapped with peaks in CTCF in the 8 cell lines in UCSC browser (Fig. 4A). Analysis of NRP1 DNA sequences from the AR-DNA binding region revealed three potential AR half binding sites and two FoxA1 binding sites. The AR half sites are shown in red in Table S6.

We next performed ChIP-PCR on LNCaP and LNsip53, with specific primers flanking these peaks. As shown in Fig. 4B, AR occupancy of the genomic region represented by the ACCA and NRP1 peaks was detected in both cell lines after DHT treatment, but was much lower in the LNsip53 cells. Occupancy of these regions by FoxA1 was observed predominantly in LNCaP, with lower levels in LNsip53. The levels of ACCA and NRP1 mRNAs increased after 8 hours of DHT treatment in the LNCaP, but did not change in the LNsip53 (Fig. 4C). This finding indicates that AR-dependent promotion of ACCA and NRP1 expression is diminished in the absence of p53.

### Analysis of LNsip53 specific AR-binding peaks

**Figure 5:**
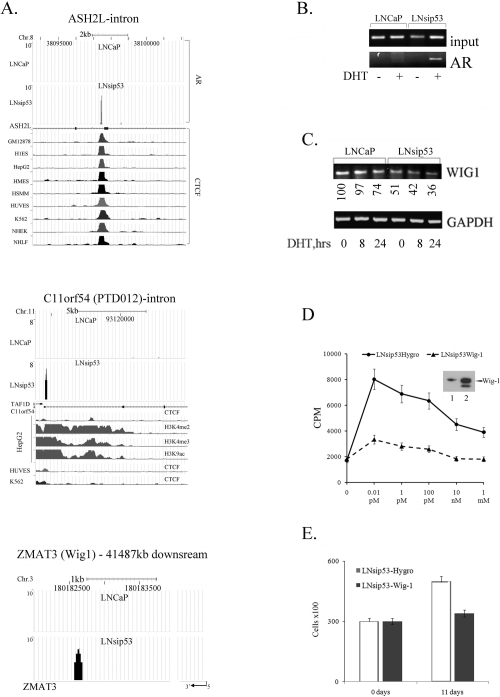
ChIP-seq analysis of LNsip53-specific AR-binding peaks (A) Visualization of AR binding to introns of ASH2L and C11orf54, and to sequences downstream of Wig-1. (B) ChIP-PCR verifying that AR binds to DNA downstream of Wig-1. Primers were derived from ChIP-seq analysis. (C) Semi-quantitative RT-PCR levels of the Wig-1 mRNA. (D) Overexpression of Wig-1 inhibits prolifiration 48 hours after DHT treatment. Cells were labeled with [^3^H]-thymidine for 16 hours prior to harvesting. Incorporated radioactivity was measured by liquid scintillation counting. Error bars represent mean ±SEM, where n=6. Western-blotting confirming Wig-1 overexpression is shown above the graph. (E) Wig-1 overexpression inhibits LNsip53 growth in the absence of androgens. Cells were incubated in CSS for 11 days, and cell number was then counted. Error bars represent mean ±SEM, where n=3.

LNsip53-specific AR-binding peaks were the most intriguing group. However the height of AR binding peaks for this group was 3 times lower on average then in LNCaP and this group had a high number of false positive peaks. For example, using the USCS genome browser we could visualize LNsip53 specific peak in an intron of ASH2L (Fig. 5A, upper panel). The DNA sequence under this peak contains a FoxA1 motif and also co-localized with a CTCF-specific peak in all cell lines represented in the genome browser. Another interesting peak was found in the first intron of chromosome 11 open reading frame 54 (C11orf54) (Fig. 5A, middle panel), also known as the PTD012 gene. This peak contains an AR half site motif and FoxA1 motif (Table S6), and co-localized with H3K4me2, H3K4me3, H3K9ac in HepH cells. However, we could not verify these peaks by ChIP-PCR.

Most notably, we found an AR binding peak downstream of Wig-1 (Fig 5A, bottom panel), a gene whose expression is known to be dependent on p53. Although we could not identify AR or FoxA1 response elements in this region, ChIP with an AR antibody followed by PCR with primers specific for AR validated that AR binds the region downstream of Wig-1. Also, the PCR product was observed in LNsip53 DHT-treated samples, but not in LNCaP control samples (Fig. 5B). ChIP using the FoxA1 antibody and the same primers failed to produce a band, and semi-quantitative PCR revealed that levels of the Wig-1 mRNA were lower in LNsip53. Semi-quantitative RT-PCR revealed that in LNCaP, DHT treatment led to a decrease in the levels of this mRNA after 24 hours, and in LNsip53, to a decrease at 8 hours and a further decrease by 24 hours (Fig. 5C). To assess the role of Wig-1 in p53-deficient cells, we cloned human Wig-1 into LNsip53 cells and estimated [^3^H]-thymidine uptake in those cells after treating them with DHT. As shown in Fig. 5D, incorporation of [^3^H]-thymidine was much higher in LNsip53-Hygro cells than LNsip53-Wig-1 cells, as measured after treatment with DHT at various concentrations. We also measured cell proliferation in these cell lines by carrying out cell counts after culturing them in CSS for 11 days. As can be seen in Fig. 5E, Wig-1 overexpression inhibited cell proliferation in CSS. Thus, expression of the Wig-1 mRNA is regulated by androgens in the absence of p53, and Wig-1 overexpression in LNsip53 cells inhibits their proliferation. This observation suggests that Wig-1 plays an important role in CRPC progression.

## DISCUSSION

The critical role of p53 in tumor development is well appreciated, but its role in PC is less clearly defined. Alterations in the p53 tumor suppressor are clearly associated with progressive disease, including “androgen-independent” growth of PCs and metastases to bone [6, 8, 23]. Loss of chromosome 17p, on which p53 is located, occurs with moderate frequency in advanced PC, and loss of one allele accompanied by point mutations in the remaining copy of p53 leads to functional inactivation (reviewed in [24]). In contrast to wild-type p53, the mutant protein can be stably expressed, and detection of high levels by immunohistochemistry is of prognostic significance. Our data indicate that inhibition of p53 promotes the proliferation of PC cells in the context of androgen deprivation. Future studies elucidating the details of the mechanism underlying p53's influence on CRPC growth are expected to lead to the identification of new molecular markers and therapeutic targets. Increasing evidence suggests that p53 directly regulates androgen signaling via AR, and that this regulation can exist at multiple levels due to the functional complexity of both proteins. It was previously suggested that the presence of wild-type p53 at basal physiological levels is necessary for AR signaling [25]. In the context of this background, our findings support the concept of a need for balance between AR and p53 expression during androgen-dependent cell proliferation, and highlights the importance of p53 as an AR modulator in prostate cancer.

Our investigation of the role of p53 in AR signaling in PC took advantage of LNsip53 cells, which had been generated in our laboratory and were described previously [10]. In support of previous work [8], we observed that LNsip53 can proliferate in the absence of hormones (i.e., in CSS). In CRPC, AR activity is thought to be maintained abnormally through several mechanisms, including AR amplification, AR mutation, an increase in AR sensitivity, local androgen production, and growth-factor activation [2, 26, 27].

Since eliminating p53 did not affect the binding of AR to specific short DNA sequences *in vitro* (EMSA, Fig. 1C) but affected occupancy of the PB promoter region by AR and FoxA1 (Fig. 1D), we suggest that p53 elimination affects binding of AR to DNA at the chromatin level. Indeed, analysis by ChIP-seq revealed differences in the binding of AR to chromatin in LNCaP vs. LNsip53. In general the LNsip53 peaks were less numerous and of lower amplitude, and a lower percentage of LNsip53-specific peaks had the expected AR motifs.

The AR-specific peaks found in LNCaP are in a good agreement with those reported previously. In spite of the fact that we found fewer peaks than Yu et al. [21], probably due to our use of a monoclonal anti-AR antibody, 75% of these matched peaks found in the earlier study. Also, our finding that AR is preferentially recruited to non-promoter-regulatory elements is consistent with earlier findings [17, 19].

The fact that our ChIP-PCR validation was less successful for the LNsip53 peaks than for those in either the LNCaP-specific or shared groups suggests that some of these may either be transient or represent false positives – and thus may not be relevant. Further studies looking at additional samples, as well as analyses of peaks with putative AR binding sites, will be required to enumerate the true LNsip53-specific AR binding sites. Nevertheless, some validation was successful, a particularly notable example being our demonstration that Wig1 expression is reduced and that this has functional consequences. This finding supports the hypothesis that AR binding in the absence of p53 is physiologically relevant. Our further finding that Wig-1 overexpression suppressed the proliferation of LNsip53 cells partially explains why LNsip53 proliferation occurs in the absence of androgens, and supports the notion that p53 plays an important role in the progression of PC to a castration-resistant state.

**Figure 6:**
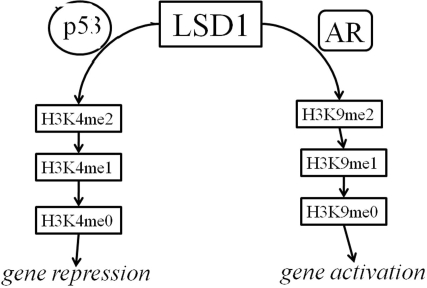
Schematic depicting model for LSD1 regulation by p53 and AR When LSD1 interacts with oligomerized p53, H3K4 is demethylated and this leads to a repression of target-gene expression (left branch). When LSD1 interacts with dimerized AR, H3K9 is demethylated and target genes are activated (right branch). In normal prostate cells there is likely a balance between the two pathways, but in the context of the many CRPCs with reduced p53 function, the right branch would be overactive.

Functional FoxA1 sites were identified in the PB and PSA promoters, as well as other prostatic enhancers, across a range of species, some of these sites were immediately adjacent to AREs, suggesting that the organization of cis-acting elements is well conserved, and that FoxA1 plays a fundamental role in prostate-specific gene expression. A direct interaction between FoxA1 and AR has been observed in these promoters, suggesting that these transcription factors cooperate in promoting the expression of androgen-regulated and prostate-specific genes [28]. The binding of DNA by FoxA1 has been reported to disrupt nucleosome formation, and thus to contribute to chromatin-mediated transcriptional repression [29]. We speculate that elimination of p53 changes the histone signature around AR-specific genes, directly affecting the priming of regulatory regions by FoxA1 binding and, consequently, binding of AR to the DNA. Several lines of evidence lead us to suggest that p53-regulated histone methylation involves lysine-specific demethylase 1 (LSD1). This demethylase co-localizes with the AR in the normal prostate as well as in prostate cancer, interacts with AR *in vitro* and *in vivo*, and stimulates AR-dependent transcription: LSD1 inhibition abrogates androgen-induced transcriptional activation and cell proliferation [30]; LSD1 forms a chromatin-associated complex with ligand-bound AR; LSD1 relieves repressive histone marks by demethylating histone H3 at lysine 9 (H3K9) (Fig. 6); and, complexes containing both p53 and LSD1 have been shown to regulate transcription in a gene-specific manner [31]. Thus, elimination of p53 may exert its downstream effects on AR-mediated transcription by interfering with LSD1 activity and the histone methylation profile across the genome.

In conclusion, our findings suggest that reduced p53 expression, which is frequently observed in CRPC cases, modifies the specificity of chromatin binding by AR, thereby leading to changes in AR-mediated signaling. This finding might be important for clinical prediction of CRPC development. Molecular markers are not routinely used to evaluate patients with PC because their relevance to important health-related outcomes has not been well defined. However, results of Concato et al., 2009 [32] suggest that p53 dysfunction is associated with increased risk for death from prostate cancer. Taken together our data suggest that understanding the p53 status is important for managing patients with CRPC.

## METHODS

### Cell lines, reagents and estimation of proliferation

The human prostate cancer cell line LNCaP was obtained from American Type Culture Collection, and the C4-2B cell line was purchased from ViroMed Laboratories, Inc. (Minnetonka, MN). Both cell lines were cultured in RPMI 1640, as previously described [10]. To culture cells under steroid-free conditions, RPMI 1640 was supplemented with 10% charcoal stripped serum (CSS) (Hy Clone, Logan, UT, USA). The expression of endogenous p53 in the LNCaP, C4-2B and Rv1 cell lines was inhibited by infection with a recombinant lentivirus construct pLSL-puro expressing an siRNA hairpin against p53 or E6 (control), as previously described [10], resulting in the LNsiE6, LNsip53, C4-2BsiE6, C4-2Bsip53, Rv1siE6 and Rv1sip53 cell lines. For estimation of cell growth LNCaP, LNsiE6, LNsip53 or LNsip53Hygro and LNsip53-Wig1 were plated in 24-well plates at a concentration of 3×10^4^, in triplicate in CCS. Cells were counted 10 days later using trypan blue to assess cell viability. The results for LNCaP and LNsiE6 did not differ and therefore only one control is shown in each Figure.

### Luciferase-reporter assay for AR activity

The luciferase reporter vector (pARE-Luc) was provided by Dr. Katerina Gurova (Roswell Park Cancer Institute, Buffalo, NY, USA) and consists of a cassette containing three AREs from the rat probasin promoter followed by an Hsp70 minimal promoter; it produces almost zero background expression in AR-negative PC cells. Cell lines possessing an integrated ARE-Luc reporter construct were generated by transfecting cells with pARE-Luc and then selecting them using G418. Luciferase activity was measured using a luciferase assay kit (Promega). The values of the luciferase assay were normalized with respect to the values of the protein concentration.

### Western blot analysis

Western blot detection of proteins was performed as described previously [33], using mouse monoclonal anti-p53, anti AR, (Oncogene, Uniondale, NY, USA), anti-Wig-1 (GeneTex, Irvine, CA, USA).

### Chromatin-immunoprecipitation and sequencing (ChIP-Seq)

LNCaP or LNCaPsip53 cells were cultured in CSS for 3 days and treated with 10 nM DHT for 1 hour. Samples for Solexa ChIP sequencing (ChIP-Seq) were prepared using ChIP IT Express Magnetic Chromatin Immunoprecipitation Kit (Active Motif, Carlsbad, CA, USA). As recommended by the manufacturer's instructions, 10 μg of DNA were used for each IP probe and incubated with 2 μg of anti-AR mAbs overnight at 4°C. Immunoprecipitations were verified by PCR with primers specific for a known AR regulatory region of the KLK3 (PSA) promoter and enhancer described in Figure S3, and 200 ng of DNA were submitted for ChIP-Seq analysis to the DNA facility of the Iowa State University (ISU) office of Biotechnology.

The results of ChIP-Seq were obtained using anti-AR or anti-FoxA1 (Santa Cruz Biotechnology, inc., Santa Cruze, CA, USA) antibodies as described above, followed by PCR with primers specific for AR binding regions derived after analyzing ChIP-Seq results. (For PCR primers sequences see Table S7)

### ChIP-Sequence data analysis

ChIP was performed with anti-AR as described above. Six ChIPs corresponding to three batches of chromatin from DHT-treated cells were pooled for a single round of Solexa sequencing. Fastq files were acquired from ISU and aligned with ELAND extended. The fastq files were first converted to aln files using a simple script to rearrange the data columns. These files were then analyzed using cisGenome, with the following settings: width = 100, step size = 25, and a cutoff of 5. The .bar ouput files from this process were converted to .txt files using the program affy_bar2txt (supplied by cisGenome). These .txt files where then converted to .wig files for uploading to the UCSC genome browser for manual viewing. The .cod files output from the same process as the .bar files were converted to .bed files using the program cod2bed (supplied by cisGenome). Partek GS (Partek Inc St Louis MO) was also used for peak prediction. Eland-aligned sequences were analyzed using default settings and cutoffs. These peaks were compared to the cisGenome peaks, and in general good agreement was found. However, no statistical test was possible due to lack of replicates. The results from Partek were used to generate .bed files, which were uploaded to the cis-regulatory element annotation system (CEAS) for processing, to find enriched regions and motifs [20], (Web Server issue):W551-4. CEAS: cis-regulatory element annotation system). An additional script was developed to extend the data from CEAS, adding additional sequence information as well as adjacent gene information based on the motif files from CEAS. This script queried the UCSC genome database for gene information, and used the alignments reported from CEAS to obtain adjacent sequence information.

The Database for Annotation, Visualization and Integrated Discovery (DAVID) was used to identify functional pathway analysis [34, 35]. The short-read sequencing data have been deposited in the Short-Read Archive, under accession number GSE31294.

### Electromobility shift assay (EMSA)

LNCaP and LNsip53 cells were cultured in CSS for 3 days, and were then treated with 10 nM DHT for 1 hour. Nuclear proteins were isolated using NE-Per nuclear and cytoplasmic extraction reagents (Pierce Rockford, IL, USA), and EMSA was performed as described earlier [36]. Double-stranded AR response element oligonucleotides [36] (or TTRs – consensus FoxA binding sequence oligonucleotides) [18] (IDT, Coralville, IA), were used as probes to detect DNA binding.

### Semi-quantitative PCR

Cells were cultured in CSS medium for 3 days and treated with 10 nM DHT for 4, 8 or 24 hours. RNA was purified using the RNeasy Mini Kit (Qiagen). Equal amounts (1 μg) of total RNA from control and treated cells were reverse transcribed using the iScript cDNA Synthesis Kit (Bio-Rad, Hercules, CA), and the cDNA (5 μL) was amplified with primers specific for selected genes (for primers used in quantitative PCR see Supplemental Table 3). PCR was carried out in the linear range for each transcript, and compared GAPDH was used as a reference control. The PCR products were analyzed on 2% agarose gels. Ethidium bromide-stained gels were scanned on a UPV bioimaging system, and analyzed using the LabWorks 4.6 software (UPV, Inc, Upstate, CA, USA). Negative RT-PCR controls were done in the absence of RNA

### Wig-1 cloning

A Wig-1 was made with PCR primers based on the mRNA sequence of *Wig-1* from *Homo sapiens* (NM_022470), and cloned into the expression vector pcDNA3.1/Hygro (Invitrogen, Carlsbad, CA, USA). Lipofectamine was used for transfection, according manufactures instructions. Empty vector was used to generate control cells resistant to hygromycin.

### Proliferation assay

The proliferation assay was carried out as previously described [33].

### Statistical analysis

Statistical analysis was performed using the Student's t-test. The statistical significance was determined at p < 0.05. Columns and points in Figures 1, and 5 show mean values for four replicates in one of three separate experiments, all of which gave similar results; error bars represent the standard error of the mean (SEM). Columns in Figure 4 show mean values for 3 separate experiments; error bars represent standard deviation (SD).

## Supplementary Tables, Figures and Methods




















