# Supplementary material for: Inhibition of p53 expression modifies the specificity of chromatin binding by the androgen receptor
Source: Oncotarget. 2012 Feb 29;3(2):183–94. doi: 10.18632/oncotarget.449 (PMC3326648; doi:10.18632/oncotarget.449)
Supplement: Supplementary file 6 [file oncotarget-03-183-s006.pdf]

**Guseva et al - Inhibition of p53 expression modifies the specificity of chromatin binding by the androgen receptor**

**Table S6. DNA sequences under chosen peaks**

Possible ARE half sites are shown in red.

Specific LNCaP peaks

ACCA_Peak_1
>hg18_dna range=chr17:32753190-32753468
5'pad=25 3'pad=25 strand=-
TTCTCTCATGTGTCTTAAAACATATGTTCAAGTCACTGAATGATCTTCAT
TTTATGGTTAACATAAAATGAAGCATGTTTGGTTAACAACTTTATCTG**AG**
**AACA**ATCTGTATTTGCTGTTATATGTGTATACCTAAAACCAGCGTAAGAT
GTCTATCACTGGGGTTCAAGACAATAGTTGTTGCTTTGGAAGGAGAAAGT
ATATTCACTCAGGAAGGGCTCTGAAGTTGAGTTATATTTCTATTTCTTTT
CTGATCTAGAAAGAAAAGTACTTTACCAA

ACCA_Peak_2
>hg18_dna range=chr17:32748970-32749290
5'pad=25 3'pad=25 strand=-
GAAAAGAAAGTGCAGGAAATTTTATTCCTGCTTCCTATCTATTGGTTCAT
CACTATCAAGCTTTGACATGGGAAATAAGGAAATTGGCTCTGGTTTTGTT
TATATTAAGGCTTTGCTTATGGATAGTTTGAGAG**TGTTCT**TATGTATTGA
CTTTCTTAGCATTTTTCATTTAGCTTTCCTGAGCTTCTTCCCTTCCCCTA
ATAGCTCTCCAAACTTTCTACTTTCAGTGCTCCTTCCTTGATATTCTGAG
GTTCCCAGTTGCCATGGTGCCTTTGTAGTGAGTAGACAGAGAAGGTCATT
ATGAGATCTGCAACTTGGTAC

ACCA_Peak_3
>hg18_dna range=chr17:32746620-32746908
5'pad=25 3'pad=25 strand=-
CAGAGATTTAATCAGCCATGCCTACTTAATGAAACCTCCATAAAACCTTG
TAAACAACAGAGTTCATAGAGCTTCTGGGATGGTCAACACATAGAGGTTC
TGGTAGAGCTGTATGCCTGGAGGAAGGTTTGGAAGTTCTGTGCCCCTTCC
CCCATACCTTGCCCTATATGTCTCTTCCTGAGTTCCTGAGTTGTATCCTT
TATAAAGCAGTAATAGTAAGTAAACTCTTTTCCTG**TGTTCT**GTATGTCAT
TCTAGCAAATTATCAAACACGAGGAAGGGGTCATGGGAA

ACCA_Peak_4
>hg18_dna range=chr17:32599820-32600055
5'pad=25 3'pad=25 strand=-
CCTCTTCAAGATGAAAGGTTCTGTATAAATAAAAGCTTTCGATTTTAGTA
CATGTTTGTAATACATGT**GGTACA**GTCCTAAATATGTATTTAGCTGATAG
GCAC**TGTTCT**TGTCTATTGTACAAATATTGACTTTAAAAATATAAATATG
TATTTAACCATCAGACCAGAAAAACACTGAAGCAGTTACTTCTGAGGTAG
AGACATTAATATTGTATAAACCTATATATACATATG 32599820

NRP1_intron peak
>hg18_dna range=chr10:33523139-33523405
5'pad=25 3'pad=25 strand=-
TGGTATCATCAGAATATGAAGAGCCAAAAGAATGGATGATGTCATTTTCT
GTTCTATAAACAATAAATGTAATGTATTAACAAAGTAAATACAGGCAGAA
CTGTGTGTGCTGGGGTGGCTTTCTTTATCCTGAATGTGCATGTTTTATAA
AGTACATTTAAGGAGAAAAATCAAAACCACACACACATTAACATGTTCCA
ATATTAACATTTTCTAACATGAGATTATGCAAC**AGTACA**TTTCAGGGTCA

TGGGCGTTGGCATCCAA

Specific LNsip53 peaks

ASH2L – intron
>hg18_dna range=chr8:38097365-38097525
5'pad=25 3'pad=25 strand=+
GGGAAGATATCCACAATCTACAAGTAATTTCACCAAAACTACCTTGCTCA
ATAAAGCTAATCCTTTTCACTTCCTTGTTTTGCCTGAAGAGGGCAGAGTT
ACTTATTTGAGTAATGAGAAGTATTTGCTGTGAAGTTGCATGTATTCTAG
CTGAAATAAAC

C14orf54(PTD012)- intron
>hg18_dna range=chr11:93114527-93114701
5'pad=25 3'pad=25 strand=+
TACCGTGACCGTTTAGGTGAGTGGAATAGCCAAGAACATTTCGGCAAATA
ACAAAAACAAAACGTGCGGTCACTTGTGAGTGGATGAGAACTGTGAGGAA
ACAGCCTCTCAGCCCGGTTCGTTTCCGGTGGTCTCGCGTTTGCTGAGCTG
TCGCCTGGCTTTCTAAATCTGGTCC

PTK2- intron
>hg18_dna range=chr8:141791833-141791992
5'pad=25 3'pad=25 strand=+
AAGGGGGGAAAAAAGGAGAAAAAAAATCCACTATTTATCTTGTACTTTCAA
TGGAACTTCTACTCATGTGTGATTTTTAACATCCTGTGTAGGTCATTTGG
AAGATACTGATTTACTGAGCTATAGAGATCCTCCCAATGTTAACACATTT
CAGTAAACAA

SATB1-147671bp upstream
>hg18_dna range=chr3:18589415-18589615
5'pad=25 3'pad=25 strand=+
TTCCTAGTGCCATTTAAATCTGGGTTGGTTTTCACTAGAGCTGCAGTAGG
ATAACAGAGCAAGAAGTTTCAAAGCAGATGTATCATGCCTCACAATGCAG
GTGAGCAAGTACTTCAAGAGGTTTTGAACCTGGCTTTACCTCTGTTTTCT
AGTGCTGACTGAAAAGCAATCAATGAGACAGGCTAGTTAAGAAATGAGTA
A

CD101-intron
>hg18_dna range=chr1:117370542-117370752
5'pad=0 3'pad=0 strand=+
CAGCAGGGCTTCCAGAGGGGAAGTCGAAGCTATCCGATTTGTTCCTTGAG
GTGGTCCATCATTGTTTTTCCCTGTTCATTCCTCAGCAGAAGTTTTCTCC
TGAGTGCCAGGCCAAAATGTTTTTTCTATCAGCCTTTTTCTTCTTTCCAA
CAATTACAGGAAGTCCCATACTCATGAGCACATTCCGTACATGTTTTCCC
AGTGGAATTAA

PTPLAD1 - intron
>hg18_dna range=chr15:63613696-63613867
5'pad=0 3'pad=0 strand=+

AACTGTCCAGTCATCACATCTCTATTCTGTCTTGGATGCTGCAGGGCTAG
TGACTATGAGAGAACACGGCTGATGTGAGGAGAAATGGTGGACAATAAAG
ACTTCAGCAGTGGCATCGTCTGTGCTTGAGGTGAACGGTAGCAAAGAGGC
CAGCTAAAGCAGGGTTACTTTC

TMEM18 – 108852bp upstream
>hg18_dna range=chr2:548930-549159
5'pad=0 3'pad=0 strand=+
ATAGGGCTTCTCTGAGGAGACAAATCATTGGATTGGGCTTAGACGTTCCA
TCCGTGGCAAGGTATGAAAAACAAACAAAATGCCACCAGGTAGTAGCGTG
ACGTTCTGTCCCCGTTCCTGTCTTTGGTTTATAGCCTTTTCTTTCAGAGA
CGGAATGTTTCCGTGTTTTATATCCACAGATTTCCGGCTTTGCCCTTTGG
AGCCTGAGGAGCAAGGGTAGAATGAGGATC

ZNF 217 Region starts 36663bp after
>hg18_dna range=chr20:51669645-51669832
5'pad=25 3'pad=25 strand=+
CTTTTTTTTCCCTTCTGCTTTAAAAATAAGATAAGCTTGCATGCAAATAG
AGTTCAGGGTGACCACAGTAACATTCTCAGCTTTATTTACTGAATTACGG
AGTCTCCCTTCACTTGATTAAGTTCCATTTGCATATCTTTAACATGAGAA
ACACTTTATTCCACTTTATTCCATTAATGAGTCATTTG

ZNF366-Region ends 33513bp before ZNF366
>hg18_dna range=chr5:71741266-71741588
5'pad=25 3'pad=25 strand=+
CCCTTTACATTTTTGTAACAGTGTGGGAATTAATGCAAAAAAAAAAAAAA
AAAAGAAAGATTACACAGGTGCTAGAATTCACTGCAGAGCCAACGGGGAA
GGCGAGGCAATCCAGAGTTTAGGAGTGAAGCCGCTGCCATTCCTGTGAGC
ATCAGGAGTTGTGGGCATCAGATGGGATCTAGAACCATGACAAGCCTGGT
GGTCACGAGCTGGATCTTCAGCAGTGACTTTCAGAGGTGCCACCTGAGGT
AGAGCGTGGGGACCCACCTTCTCCTTTCCTCCCATCTCCAAGCTCTATAC
CAGTGTCCCCATGGCACTGTATA

CTBP2-Region contained within
>hg18_dna range=chr10:126789585-126789750
5'pad=25 3'pad=25 strand=+
GATGGGCTGTCCGTCTTTTTAAGGGAATACACCTTCATTGGTTCAAAACC
ATTTAAGGTGATGAAACCCTGAAAGCAAAATGAATGGAGTCAGTGAAGAT
GAAGTAGAGATTTTTTTTTTTTTGTTTACATCTAGGCATCCTTTAATTGCA
TTTTTCCAATGCCTTC

TCF4-Region ends 125869 bp before
hg18_dna range=chr18:50914535-50914786
5'pad=25 3'pad=25 strand=+
TGTGTGTTTAACTAGAAATGGGGTGATCAGAGGGAGAAAGGAGGCCATGG
GGGTCTCATCACTTAACACTAATGGTGCCTCTTCCTGGGAGCTTTTGCGT
ACTCATGCCTTCATTTCTTTATGCTCATCCATGTCTCTTAAGCACGCCAA
GAAGTTTCTGAGCCCACACTAGATGGCAGCATGGTTTACTGGTCATGTGG
GCAGAGCCAAGGCCCCAGCAGCCTGAGCCCACATGATGCTCATCCTTGCT
TT

AGPAT6 Region ends 14035 bp before
>hg18_dna range=chr8:41540585-41540795
5'pad=25 3'pad=25 strand=+
TCTTACAAATTTTTATAGCACTCTTGAAAATAATTCTTGTGTCAAAGAGA
AAACAAATATGCAAATGAGAGCCTGTTTAGCAATAAAGCACTGTCTTCAA

ATGTATAGGTTTACTTTTTTGAGAGAATATACAATCTCAAATGCATTTCT
TGGGAAACACAAAAGATTAAAAATAAAGGAAACTGAGCATTCCACTGAAG
AAGGGAGAAAC

ZEPM2 Region ends 104436 bp before
>hg18_dna range=chr8:106295657-106295894
5'pad=25 3'pad=25 strand=+
ATCTCTGCAGCGTTATCCTAAGGAGTATAATTAATATTTTTCTCTTTTAA
CCATATTGCACTGGTTAAAAATTTCAGTATTAAGTTCTGTGACATAGCAG
TCATTGTTTCCTGAGACTATGGAATTGTAAATCTCACTTACTGTTCTACC
TCGTTATTAGATGCAAAAAGAAAATGTACTATCATTGACTGCAAAAGTTA
ATATTCATTTGTTGCAAGCAAAGAAAGTTGAGTATGAC

GRB10 Region starts 108206 bp after
>hg18_dna range=chr7:50875762-50875934
5'pad=25 3'pad=25 strand=+
CATATGTCAACCACAGCACAGGTGTTCATTTCAAACAGTTCCTTTCTGCT
TTTTATCCCAACTTTTTATTATAACAAATTTCAAACATATAGAAAAGTTG
AAATTATACTACAATGAACACTTGTATGTTTACCACATAGAAACAACAGT
TTTTAACAATTTGCCGTATTTGC

CCBE1 Region starts 66130 bp after
>hg18_dna range=chr18:55581713-55581897
5'pad=25 3'pad=25 strand=+
ATGAGGTTTCATAAACATAGCCTTCTGGATCCTGTTTACTTGGTTGGAAC
GCATCTAGAGGATGTGCTCCACCAAAATAAAAGAATAAGCCAAAGCTGAG
ATGGCACAGGATTTAACACAAGAGGGAGGCACAGGGCATCCTGAGCTGTT
GCTGAGGGAATATCCCAGGACCACAGCTGTGCTCA

ZMAT3 Region ends 41487bp before
>hg18_dna range=chr3:180182529-180182782
5'pad=50 3'pad=50 strand=-
TACGTGGCTATCCACTGCAATAACTGATAGGAAATTCCCCAATACTGCCT
GATACTTAATAAGTGCTTACTACATTTTCCCTGAGTGTGGCAATCAAACA
TATTGTAGACTAGCTTTTTCTCTCCCCTAATTTCCAATTATATGCTGACG
TACATTTAAACCTTATTGCATCTTAGTTTGCTTGTTTCCAGTATGTTAAT
TTAGTCAACTCTGAGAGAGTTTTAGCATGTGGTGATGGCTAAGTTTTTGT
AATA
