# Supplementary material for: Inhibition of p53 expression modifies the specificity of chromatin binding by the androgen receptor
Source: Oncotarget. 2012 Feb 29;3(2):183–94. doi: 10.18632/oncotarget.449 (PMC3326648; doi:10.18632/oncotarget.449)
Supplement: Supplementary file 8 [file oncotarget-03-183-s008.pdf]

Guseva etal - Inhibition of p53 expression modifies the specificity of chromatin binding by the androgen receptor

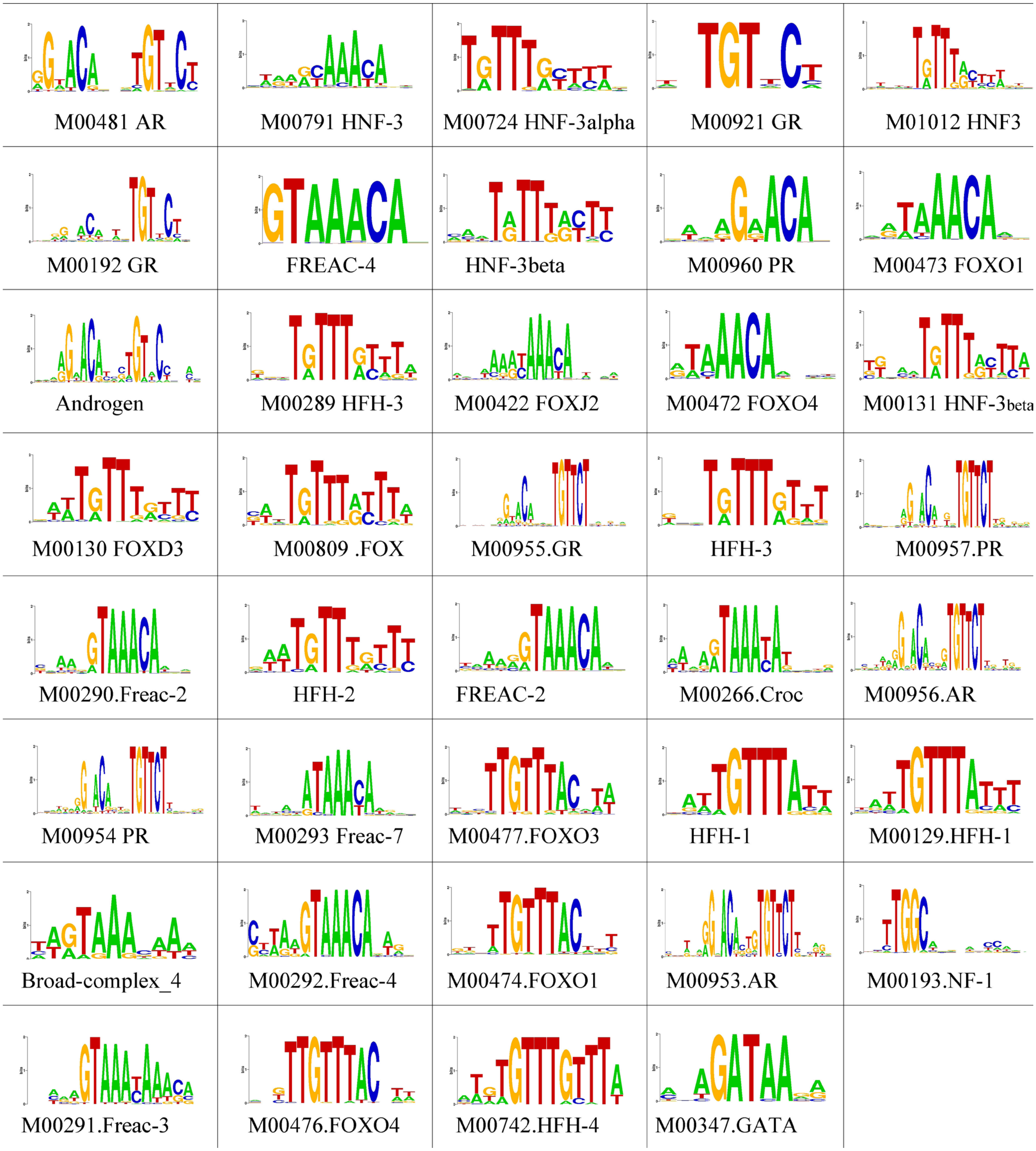

Figure 1S: LOGOS derived after motif enrichment analysis of LNCaP. The .bed files of ChIP sequences of LNCaP where uploaded to the cis-regulatory element annotation system (CEAS) for processing to find enriched regions and motifs (Web Server issue):W551-4.
