# Supplementary material for: Inhibition of p53 expression modifies the specificity of chromatin binding by the androgen receptor
Source: Oncotarget. 2012 Feb 29;3(2):183–94. doi: 10.18632/oncotarget.449 (PMC3326648; doi:10.18632/oncotarget.449)
Supplement: Supplementary file 9 [file oncotarget-03-183-s009.pdf]

Guseva etal - Inhibition of p53 expression modifies the specificity of chromatin binding by the androgen receptor

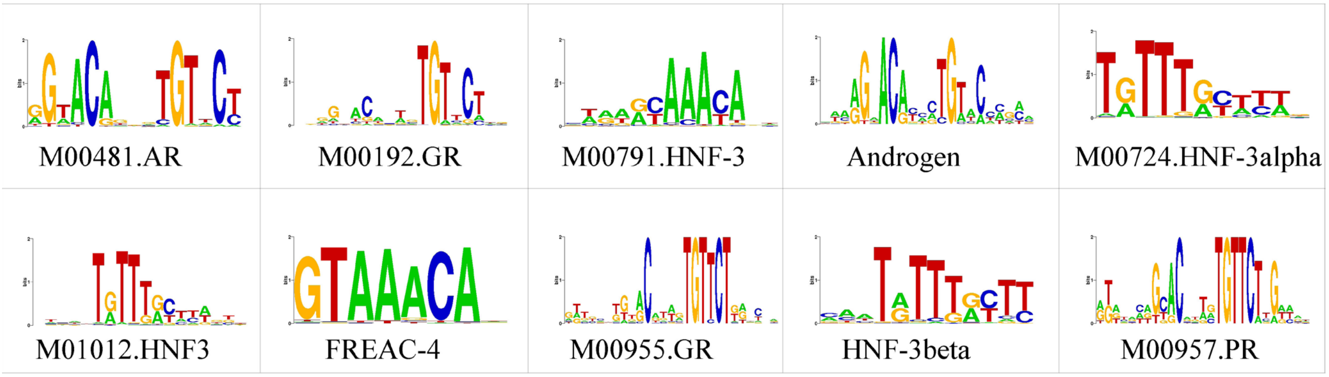

Figure 2S: LOGOS derived after motif enrichment analysis of LNsip53. The .bed files of ChIP sequences of LNsip53 where uploaded to the cis-regulatory element annotation system (CEAS) for processing to find enriched regions and motifs (Web Server issue):W551-4.

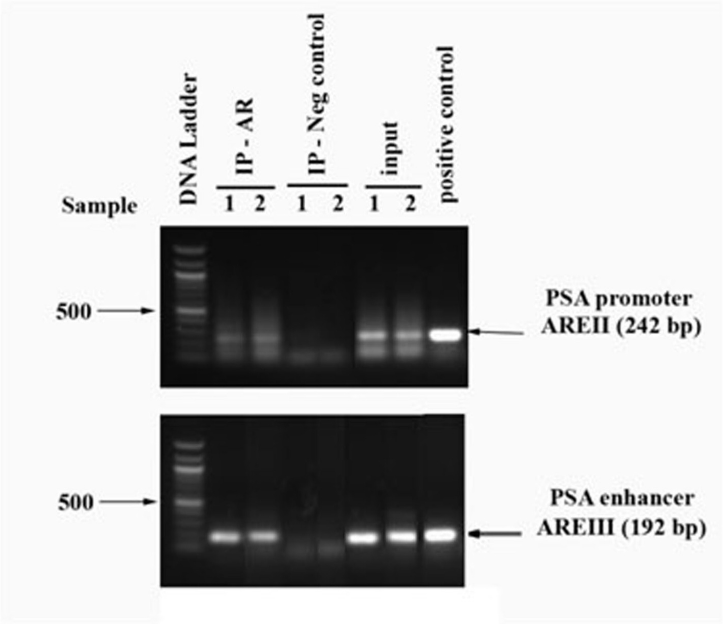

Figure 3S: ChiP-PCR of AR occupancy on the PSA gene regulatory region in response to DHT treatment. LNCaP and LNsip53 cells were cultured in CSS media for 3 days, treated with 10 nM of DHT for 1 h. Soluble chromatin was prepared from formaldehyde-cross linked and sonicated cell cultures for ChIP-Seq. analysis. To validate samples prior sequences, we verified occupancy of regulatory regions on PSA (KLK3) regulatory regions reported previously (Shang et. al., 2002). AREII located - 390 bp upstream to the PSA gene transcription start site (PSA promoter). AREIII located - 4140 bp upstream to the PSA gene transcription site (PSA enhancer). Note, negative control after IP with IgG did not amplify any fragments.
