# Supplementary material for: Inhibition of p53 expression modifies the specificity of chromatin binding by the androgen receptor
Source: Oncotarget. 2012 Feb 29;3(2):183–94. doi: 10.18632/oncotarget.449 (PMC3326648; doi:10.18632/oncotarget.449)
Supplement: Supplementary file 10 [file oncotarget-03-183-s010.pdf]

Guseva et al - Inhibition of p53 expression modifies the specificity of chromatin binding by the androgen receptor

Supplemental Documents

Inventory of Supplemental Information

Figure S1, related to Figure 2
Figure S2, related to Figure 2
Figure S3, related to Figure 2 and “Supplemental Experimental Procedures”
Table S1, related to Figure 2A
Table S2, related to Figure 2A
Table S3, related to Figure 2D
Table S4, related to Figure 3A
Table S5, related to Figure 3A
Table S6, related to Figure 4, 5.
Table S7, related to “Experimental Procedures”

Table S1. List of peaks found after ChIP –Seq analysis in LNCaP

Table S2. List of peaks found after ChIP-Seq analysis in LNsip53

Table S3. Motif enrichment analysis of ChIP-Seq data prepared by CEAS

Table 4S. List of genes determined around or within peaks by CEAS

Table 5S. List of genes with names translated by DAVID

Table 6S. DNA sequences under chosen peaks. Possible ARE half sites are shown in red.

Table 7S. Primers used for ChIP-PCR and for RT-PCR

Supplemental experimental procedures

p53 knockdown preparation

The expression of endogenous p53 was inhibited by infection with recombinant lentivirus constructs pLSL-puro-expressing siRNA hairpin under control of the RNA H1 promoter (Budanov *et al*., 2004). The structure of the 19 bp siRNA complementary to human p53 mRNA was as follows: 5′-GACTCCAGTGGTAATCTAC-3′. The structure of the control siRNA derived from the HPV18 E6 gene was as follows: 5′-CTAACACTGGGTTATACAA-3′. LNCaP was infected with lentivirus with siE6 or

si-p53 followed by puromycin selection. The effect of siRNA expression was verified by assessing p53 protein expression by Western blot analyses.

Caspase activity in cell lysates

Caspase activity in cell lysates was measured as described previously (Rokhlin et al., 2001). Briefly, cell lysates were prepared in 1% Triton X-100 buffer, pH 7.2, containing protease inhibitors. Protein lysate (40 μg) was incubated for 60 min in assay buffer (20 mM PIPES, pH 7.2, 100 mM NaCl, 10 mM DTT, 1 mM EDTA, 0.1% CHAPS and 10% sucrose) with 40 μM of fluorescent substrates Ac-DEVD-AMC or Ac-DEHC –AMC (Bio-Mol, Plymouth Meeting, PA, USA) and substrate hydrolysis was monitored using a fluorescence reading system set to 360 nm for excitation and 460 nm for emission.

ChIP

Sampels for Solexa ChiP sequencing experiment-high throughout sequencing (ChiP-Seq) were prepared using ChiP IT Express Magnetic Chromatin Immunoprecipitation Kit. (Active Motif, CA cat # 53008) following manufactures instruction with minor modifications. Briefly, cells were crosslinked with 1 % formaldehyde for 10 min at room temperature and the cross linking was inactivated by 0.125 M glycine for 3 min at room temperature. Cells were washed with cold PBS twice. Cells were harvested in cell lysis buffer (5mM PIPES pH 8.0, 5mM KCl, 0.5% NP40, PMSF and protease inhibitors cocktail) using scraper. After centrifugation, pellets were resuspended in 1ml of ice cold Lysis Buffer from ChiP IT kit and homogenized on ice with dounce homogenizer. After 10 minutes of centrifucation nuclei were resuspended in Shearing Buffer (ChiP IT kit). DNA was sheared by sonication on Sonics Vibracell sonicator at 55% power in volume 350 μl with 3mm stepped microtip. Twelve pulses of 30 second each, with a 40-second rest on ice between each pulse. 50 μl of each sample were reverse cross linked, treated with RNAs A and proteinase K according to protocol and DNA were cleaned up using QI Aquick

MINelute kit (Qiagene, Valencia, CA, USA, No 28104) and separated in 1% agarose gel. 10 μg of DNA were taken for each IP probe with 2 μg of anti-AR mAbs overnight at 4C. DNA samples were cleaned up using QI Aquick MINelute kit (QIAGEN, Valencia, CA, USA) and DNA concentrations were detected using NanoDrop 2000 (Thermo Scientific NanoDrop products Wilmington, DE, USA). Immunoprecipitations were verified by PCR with specific primers for a known AR regulatory region of the PSA promoter (AREII F 5'-AGG GAT CAG GGA GTC TCA CA-3', R 5'-GCT AGC ACT TGC TGT TCT GC-3') and enhancer (AREIII F-5'-ACA GAC CTA CTC TGG AGG AAC, R 5'-AAG ACA GCA ACA CCT TTT T-3') and shown in Figure S3.

CHIP-Sequence data analysis

Fastq files were acquired from ISU aligned with ELAND extended. The fastq files where first converted to aln files using a simple script to rearrange the columns of data. These files were then analyzed using cisGenome with the following settings; width equal to 100, step size equal to 25 and a cutoff of 5. The .bar files output from this process where converted to .txt files using the cisGenome supplied program affy_bar2txt. These txt files where then converted to .wig files for uploading to the UCSC genome browser for manual viewing. The .cod files output from the same process as the .bar files were converted to .bed files using the cisGenome supplied program cod2bed. These .bed files where uploaded to the cis-regulatory element annotation system (CEAS) for processing to find enriched regions and motifs (Ji et al., 2006), (Web Server issue):W551-4. CEAS: cis-regulatory element annotation system). An additional script was developed to extend the data from CEAS adding additional sequence information as well as adjacent gene information based on the motif files from CEAS. This script queried UCSC genome database for gene information and used the alignments reported from CEAS to obtain adjacent sequence information.

EMSA

LNCaP and LNsip53 cells were cultured in CSS for 3 days then treated with 10 nM DHT for 1 hour. Nuclear proteins were isolated using NE-Per nuclear and cytoplasmic extraction reagents (Pierce Rockford, IL, USA) following manufacture instruction. Double stranded AR response element oligonucleotides 5'-AGCTTGTCTGGTACAGGGTGTTTTTGTCGA-3' or TTRs - consensus FoxA binding sequence oligonucleotides 5'-TGACTAAGTCAATAATCAGAAG-3'(IDT, Coralville, IA) were used as probes for detection of DNA binding. Probes were end-labeled with 30 μCi of [γ^{32}P]ATP and incubated with 5 μg of nuclear extracts in a final volume of 10 μL of binding buffer [50 mM Tris-HCl (pH 7.5), 5 mM $MgCl_2$, 25mM EDTA, 1mM DTT, 20% glycerol and 1 μL of 1 mg/ml poly d[(I:C)]. Some samples were preincubated either with 100x excess of unlabeled AR/ FoxA1 response oligonucleotides or anti-AR/ anti-FoxA1 antibody as indicated. The gels (Novex 6% DNA retardation gel) were run in x0.5 TBE buffer at room temperature for 20 min at 250 V, dried and exposed to X-ray films (X-Omat, Eastman Kodak Co.).

Wig-1 cloning

A Wig-1was made with PCR primers basing on the mRNA sequence of *Wig-1* from *Homo sapiens* (NM_022470), Wig1cloneF-5'- TTTGGATCCATGATCCTCTTGCAACACGC and Wig1cloneR – 5'-GAGAATCTGGGATATGTATAGGCGGCCGCAAAAG using the Wig-1 cDNA as template. The *Hind*III/*Bam*HI restriction sites were used for cloning Wig-1 into the expression vector pcDNA3.1/Hygro (Invitrogen, Carlsbad, CA, USA). Lipofectamine was used for transfection according the manufactures instructions. Empty vector was used to obtain control cells resistant to hygromycin.

Proliferation assay

LNsip53-Hygro and LNsip53-Wig-1 were plated at 7,000 cells per well in 96 well flat bottom plates in RPMI media supplemented with 10% CSS and incubated for 3 days. Different concentrations of DHT were added and plates were incubated for 48 hours and labeled for 16 h with 1μCi/well [^{3}H]-thymidine. Cells were harvested onto filter paper, and the incorporated radioactivity was measured by liquid scincillation counting. All samples were measured in sextuplicate in two independent experiments.

Supplemental References

Budanov, A.V., Sablina, A.A., Feinstein, E., Koonin, E.V., and Chumakov, P.M. (2004). Regeneration of peroxiredoxins by p53-regulated sestrins, homologs of bacterial AhpD. Science (New York, NY *304*, 596-600.

Ji, X., Li, W., Song, J., Wei, L., and Liu, X.S. (2006). CEAS: cis-regulatory element annotation system. Nucleic Acids Res *34*, W551-554.
